# The Mixed-Method 5W2D Approach for Health System Stakeholders Analysis in Quality of Care: An Application to the Moroccan Context

**DOI:** 10.3390/ijerph16162899

**Published:** 2019-08-13

**Authors:** Youness Frichi, Fouad Jawab, Said Boutahari

**Affiliations:** 1 Laboratory of Manufacturing, Energy and Sustainable Development, High School of Technology, Sidi Mohamed Ben Abdellah University, Fez 30000, Morocco; 2 Laboratory of International Management, Decision Making and Logistics (MIDLOG), Sidi Mohamed Ben Abdellah University, Fez 30000, Morocco

**Keywords:** health system, quality of care, stakeholders, 5W, Delphi, DEMATEL

## Abstract

(1) Background: Quality of care (QC) is not only about satisfying patients, but also about satisfying the various health system stakeholders (HSS). This makes it a complex and difficult objective to achieve. This study aims at proposing a methodological framework for identifying HSS, prioritizing them in QC, and analyzing their interrelationships. (2) Methods: The proposed framework is the mixed-method 5W2D approach, which uses a combination of three basic methods: the 5W questioning technique (What, Who, Why, Where, and When), the Delphi method, and the Decision making trial and evaluation laboratory (DEMATEL) technique. It consists of three interdependent phases. First of all, a preliminary list of HSS is established based on a systematic literature review, which is then projected and adapted to the national context using the 5W questioning technique. Secondly, the identified HSS are classified in order according to their influence and impact on QC by employing Delphi method. Thirdly, the interrelationships between HSS are determined and analyzed by applying DEMATEL technique. An application of 5W2D is conducted in the Moroccan context as its health system involves a wide range of stakeholders. (3) Results: Results defined 17 groups of HSS, whose prioritization led to three groups that are at the core of the health system: patients and their families, health personnel, and government. Roles and expectations of these groups regarding QC are divergent and contradictory, which require making trade-offs. The findings of this study intend to guide the development of inclusive strategies and policies that involve key stakeholders for QC assessment and improvement.

## 1. Introduction

Quality issues have grown to be a major concern for almost all organizations; quality improvement can help at reducing costs and delays, as well as promoting the organization’s image and reputation. In the health sector, healthcare facilities are required to improve the quality of care (QC), which should be developed to best fit the general standards and regulations and meet the patients’ needs [1]. QC is a complex concept because of the multitude of stakeholders who play essential roles in the inputs, processes, and outputs of healthcare services. It is influenced by the stakeholders who make up the health system. Its assessment should consider the perspectives of the service recipients (patients), as well as the direct and indirect healthcare service providers (health professionals and managers) [2]. In this respect, Leviton and Melichar [3] claim that improving QC requires taking into consideration the perspectives of stakeholders that are essential for planning, implementing, and assessing QC improvement programs. The consideration of stakeholders’ concerns is reflected in the concept of total quality, which has been adopted in several sectors as a quality improvement approach with a client–supplier orientation. Its objective is to satisfy internal and external stakeholders [4]. The transposition of the total quality concept into the health sector has, therefore, placed the satisfaction of all stakeholders as a primary objective of healthcare facilities [5]. It is then necessary to identify these stakeholders and meet their expectations. However, health systems are characterized by the multiplicity of stakeholders who are involved in medical and logistical activities [6]. This makes achieving their satisfaction a difficult objective to realize. Meanwhile, it is of great importance to meet the expectations and interests of key stakeholders since their dissatisfaction may lead to undesired outcomes, such as: reduced budgets, loss of positions, decreased effectiveness, creating roadblocks, etc. [7]. Consequently, healthcare facilities should rank their stakeholders in order to retain the most critical ones. The selection process can be viewed as a multi-criteria decision-making problem. To ease out the process of identifying and selecting key stakeholders, an effective approach is called into action. In this article, the objective is to propose an approach for identifying health system stakeholders (HSS) and their roles, prioritizing them according to their importance in the QC, and analyzing their interrelationships. In doing so, the roles and responsibilities of each HSS would become clear, which is the first step towards improving the QC. The proposed approach is a combination of three basic methods: the 5W questioning technique, the Delphi method, and the Decision making trial and evaluation laboratory (DEMATEL) technique. The 5Ws refer to five fundamental questions: What is the topic? Who is involved? Why does it happen? Where does it take place? and When does it take place? Answers to these questions are necessary in information gathering, as well as in understanding the situation and the context. In this study, the 5W questioning technique is used to gather information about HSS and their role. Who are they and why are they considered as stakeholders? Delphi is a qualitative research method frequently used in social science disciplines. It relies on gathering opinions from a panel of experts to produce a collective agreement on a given issue [8]. Using this method, we could rank HSS according to their importance in the QC. DEMATEL is an effective technique for analyzing complex systems and developing causal relationships between its components [9]. Deploying the DEMATEL technique would make it possible to determine and analyze HSS interrelationships, and then to visualize the most influential and influenced HSS groups. We call the combination of these three methods the “5W2D”, which is an acronym composed of their initials.

The theoretical framework of the following article is based on the stakeholder theory. This theory constitutes the theoretical basis for the strategic management of organizations, which enables them to understand, associate and integrate their environment. The remainder of this paper is organized as follows. The second section consists of a general background related to QC and stakeholder theory. The third section describes the working methodology of 5W2D. The fourth section is dedicated to an empirical application of the proposed methodology in the Moroccan context. Finally, the results are discussed in the fifth section.

## 2. Background

### 2.1. Quality of Care (QC)

The main concern of this background section is to list some definitions of QC along with its dimensions in order to highlight the complex nature of quality in healthcare. The QC has been largely defined in the literature. Still, no standardized definition is agreed upon. Donabedian [10] defined QC as “the kind of care that is expected to maximize an inclusive measure of patient welfare after one has taken into account the balance of expected gains and losses that attend the process of care in all its parts”. Two aspects of care are highlighted by this definition, namely, losses with reference to safety and welfare with reference to satisfaction. The United States National Academy of Medicine (NAM) defined QC as “the degree to which health services for individuals and populations increase the likelihood of desired health outcomes, and are consistent with current professional knowledge” [11]. This definition emphasizes the effectiveness of care and the notion of probability that recognizes the uncertainty of the outcomes. Other definitions of QC exist; they are properly presented in the article by Nylenna et al. [12], who concluded that there is no universal definition that can be attributed to the concept of QC.

The variety of definitions of QC proposed in the literature makes it a difficult concept to assimilate. In order to better understand this concept, some researches assume that a health system is compelled to satisfy certain criteria so as to be considered as providing QC. These criteria are also known as dimensions of QC. The number, designations, and meanings of these dimensions vary in the literature. In this paper, we have retained six dimensions by WHO [13]. To help grasp these dimensions, we have identified for each of them some observable indicators extracted from The United States National Academy of Medicine (NAM) [14] and The Organization for Economic Co-operation and Development (OECD) [15] reports (Table 1).

Most of studies on QC have focused on two main questions: How can QC be measured and evaluated? and How can QC be improved? The purpose of the QC assessment is to improve healthcare outcomes. In this regard, patient satisfaction is considered as the most important indicator of QC. Nevertheless, some authors noted the limitations of this indicator, in particular its subjectivity and the lack of patients’ skills to judge the technical QC [16]. It was suggested that the views of health professionals should be taken into account [17], as well as compliance with standards and regulatory references. Studies related to QC improvement can be summarized in three areas: improvement of technical aspects of QC (drug effectiveness, surgery, etc.), improvement of service delivery by strengthening the performance of logistics activities (appointment scheduling, hotel services, transportation, etc.) [18], and improvement of the managerial aspects related to strategies and policies of managing healthcare services. All these improvement areas require the effective engagement of stakeholders, which is a vital condition for success [19].

### 2.2. Stakeholder Theory

The key idea of stakeholder theory is that the success of an organization relies on the cooperation with its stakeholders [20]. A stakeholder is “any group or individual who can affect or is affected by the achievement of the firm’s objectives” [21]. Thus, the organization has to expand its responsibilities to include the interests and rights of all stakeholders in order to win their support [22].

Stakeholder analysis is the process of identifying, categorizing and prioritizing stakeholders for their involvement in decision-making processes [23]. Several researchers have proposed several criteria to identify stakeholders. Mitchell et al. [24] proposed *power*, *legitimacy,* and *urgency* criteria. Kochan and Rubinstein [25] contemplated that a stakeholder has to provide resources to the organization, be affected by its decisions, and have control over it. Fritz et al. [23] suggested using the supply chain approach to identify all stakeholders involved in the product manufacturing from the raw material to the final product. Despite the existence of several approaches for stakeholders’ identification, none of them guarantee a complete identification without omission.

Various stakeholder categorizations can be found in the literature. One well-known categorization distinguished between primary stakeholders who are in direct contact with the organization and form a partnership with it, and secondary stakeholders who are impacted by the organization’s action, but in the absence of any contractual link [26]. Other categories are suggested: influencers and claimants [20], internal and external [27], etc. These different categorizations are said to be classic and generic, new ones that consider the nature of the addressed issues are required.

It can be drawn from the literature that several reasons are behind the analysis of HSS, including:

- The importance of stakeholders’ participation and involvement in the processes of identification, understanding, and resolution of health issues. Ng et al. [28] sought HSS viewpoints to spot the multiple dimensions and parameters to be included in the development of a medication safety assessment framework. Ong et al. [29] conducted interviews with HSS to identify challenges, opportunities, and ways forward for the implementation of regional health systems. Franco-Trigo et al. [30] conducted a stakeholder analysis of community pharmacy services for the prevention of cardiovascular disease. The authors of these studies highlighted the importance of involving HSS in planning processes to address health programs implementation challenges.
- Understanding and considering the different perspectives of HSS in a project decision-making process ensures its success and survival. Hamilton et al. [31] suggested that stakeholder engagement is needed to support the implementation of clinical initiatives. For instance, the design and development of new medical devices and equipment require HSS consultation to take into consideration their specific needs and guarantee their acceptance of the proposed devices [32].
- Highlighting the contradictory interests of HSS. Achieving the organization’s objectives usually involves conflicts of interests between stakeholders who do not have the same objectives [20]. Patients want effective care, health professionals demand favorable working conditions, managers seek to reduce costs, product makers are interested at defending their commercial interests, etc. [33]. Thus, it is important to maintain balance between these competing interests.

## 3. Methods

**Figure 1:**
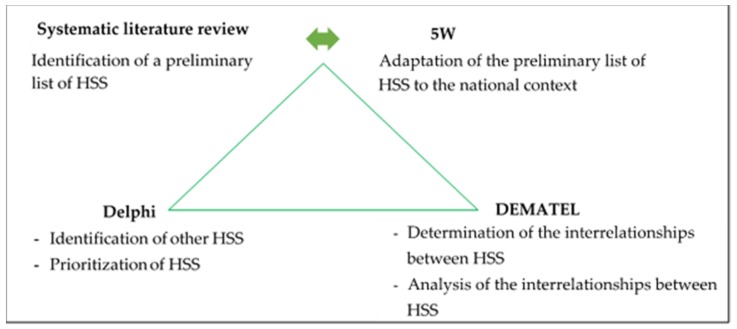
Methodological framework 5W2D for health system stakeholders (HSS) analysis.

The proposed methodological framework for identifying HSS, prioritizing them in QC, and analyzing their interrelationships is composed of three interdependent phases (Figure 1). It is the combination of a systematic literature review reinforced by the 5W questioning technique (What, Who, Why, Where, and When), the Delphi method, and the DEMATEL technique. We called this mixed method “5W2D”. It is an acronym formed from the initials of these three methods (5W, Delphi, DEMATEL).

### 3.1. Systematic Literature Review and 5W

The objective of a systematic literature review is to establish a preliminary list of HSS from previous studies conducted at the international level. Then, in order to project and adapt this preliminary list to the national context, we propose applying 5W questioning. Answering these questions allows for identifying HSS and their roles at the national level. It should be noted that the 5W has received a great attention in health research areas thanks to its effectiveness in information gathering and identifying the causes of problems [34,35,36].

### 3.2. Delphi

The aim of using the Delphi method is twofold: to complete the list of HSS established in the previous phase, and to prioritize them according to their degree of influence on and impact by the QC. A key advantage of the Delphi method is its openness to expert opinions as a source of information. Other methods of consulting experts such as the Nominal Group Technique can be used. However, the choice of the Delphi method is justified by the fact that it guarantees anonymity and the absence of interactions between experts. Also, it does not require experts meeting that would be difficult to establish, unlike the Nominal Group Technique in which the risk of influence is present and experts meeting is required [37]. In addition, the Delphi method is commonly used in ranking issues in a given field [8], which is similar to our objective of prioritizing HSS.

**Figure 2:**
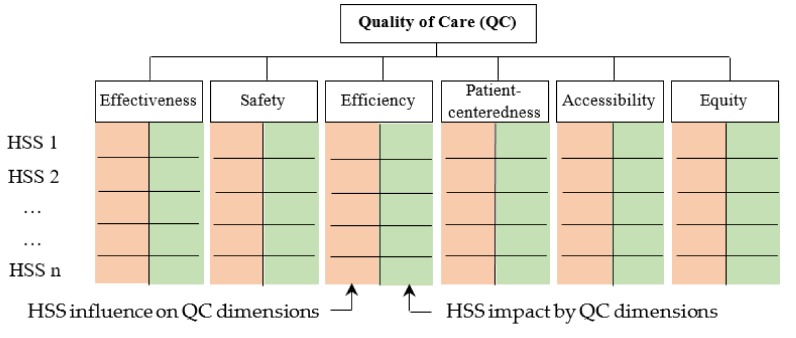
Grid for prioritizing HSS in QC.

The following steps outline how to undertake a Delphi study [38]:

(1) Identification of experts: they should belong to different disciplines (i.e., academics, practitioners, government officials) and have relevant knowledge and experience in their respective fields. We suggest following expert identification steps by Okoli and Pawlowski [39].
(2) Questionnaire design: the questionnaire contains two main questions. The first one invites experts to consult the HSS list and add others, if any, that they may consider relevant. The second one asks experts to assign two scores to each HSS regarding their influence on and impact by QC dimensions (Figure 2), using a scale from 1 to 5: “very low (1)”, “low (2)”, “medium (3)”, “high (4)”, and “very high (5)”. Table 1 about QC dimensions is appended to the questionnaire so that experts have a common understanding of the different dimensions.
(3) Consultation of experts and result analysis: the questionnaire can be administered by e-mail or by post. Before analyzing the results, expert’s responses are completed by calculating the sum of the scores assigned to each HSS with respect to its influence on and impact by QC dimensions. Let us note,
  *d*: QC dimension index (*1 ≤ d ≤ 6*)
  *E*: number of experts
  *e*: expert index (*1 ≤ e ≤ E*)
  *n*: number of HSS
  *k*: HSS index (*1 ≤ k ≤ n*)
  *M_e,k,d_*: rating given by the expert *e* to the influence of HSS_k_ on the QC dimension *d*
  *N_e,k,d_*: rating given by the expert *e* to the impact of HSS_k_ by the QC dimension *d*
  *M_k_*: the average score of the influence of HSS_k_ on QC
  *N_k_*: the average score of the impact of HSS_k_ by QC

For the expert *e*, the influence of HSS_k_ on QC is $\sum_{d=1}^{d=6}M$*_e,k,d_* and its impact by QC is $\sum_{i=1}^{i=6}N$*_e,k,d_*. The consolidated result of the expert panel is obtained after the calculation of the average scores *M_k_* and *N_k_*:

(1) $$M_{k}\;=\;\frac{\sum_{e=1}^{e=E}\sum_{d=1}^{d=6}M_{e,k,d}}{E}$$

(2) $$N_{k}\;=\;\frac{\sum_{e=1}^{e=E}\sum_{d=1}^{d=6}N_{e,k,d}}{E}$$

The degree of consensus among experts is assessed using Kendall’s *W* coefficient of concordance. For values of *W* less than 0.7, the questionnaire should be sent back to experts for new answers [39].

### 3.3. DEMATEL

In order to determine and analyze *HSS* interrelationships, we propose applying Decision making trial and evaluation laboratory (DEMATEL) technique. It is a structural modelling approach, used particularly for the analysis of cause-and-effect relationships between the components of a complex system. It classifies them into cause group and effect group, and allows identifying the critical ones [40]. It has been applied in different research areas to support decision-making: textile and apparel industry [41], electronics sector [42], sustainable development [9], energy [43], logistics [44], etc. In health science, DEMATEL was used with different perspectives: to select optimal healthcare waste treatment technologies [45], to determine key success factors of service quality in hospitals [46], and to identify critical factors that influence the emergency management process [47], etc.

**Figure 3:**
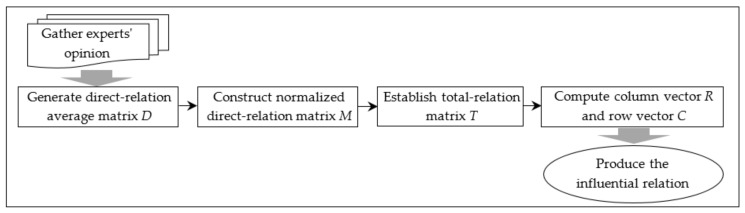
Steps to implement the Decision making trial and evaluation laboratory (DEMATEL) technique.

The DEMATEL technique can be undertaken following steps in Figure 3 [40].

- Step 1: generate the direct-relation average matrix D

Suppose there are *n HSS* and *E* experts are asked to indicate the direct influence that each *HSS_i_* has on *HSS_j_*, using a scale from 0 to 4: “no influence (0)”, “low influence (1)”, “medium influence (2)”, “high influence (3)” and “very high influence (4)”. For each expert *e*, an individual direct-relation matrix is generated D*_e_* = [d_ij_*^e^*]*_n_*_×*n*_. the d_ij_*^e^* represents the judgment of expert *e* on the degree to which *HSS_i_* affects *HSS_j_*. DEMATEL does not require a large number of experts, numerous studies have used a sample of three experts [42]. The direct-relation average matrix *D* is obtained by aggregating the E experts’ opinions:

*D* = *[d*_*ij*_*]*_*n* × *n*_   *i*,*j* = *1*, *2*, …,*n* (3)

(4) $$d_{ij}\;=\;\frac{1}{E}\times \sum_{e=1}^{E}d_{ij}^{e}$$

- Step 2: construct the normalized direct-relation matrix M

This matrix is obtained by applying Equations (5) and (6):

*M* = *u.D* (5)

(6) $$u\;=\;\min\left\{\frac{1}{max\;1\leq i\leq n\sum_{j=1}^{j=n}d_{ij}}\;;\;\frac{1}{max\;1\leq j\leq n\sum_{i=1}^{i=n}d_{ij}}\;\right\}$$

- Step 3: establish the total-relation matrix T

Once the normalized direct-relation matrix *M* is obtained, the total-relation matrix *T* can be established through Equation (7), in which *I* is the identity matrix.

*T* = *M (I* – *M)^-1^* (7)

- Step 4: compute the column vector R and row vector C

*R* is a column vector and *C* is a row vector representing the sum of rows and columns of total-relation matrix *T*. They are computed using the following equations:

(8) $$R\;=\;[r_{i}{]}_{n\times 1}\;=\;\left[\sum_{j=1}^{j=n}t_{ij}\right]$$

(9) $$C\;=\;[c_{j}{{]}^{’}}_{1\times n}\;=\;\left[\sum_{i=1}^{i=n}t_{ij}\right]$$

*r_i_* and *c_j_* are the sums of the *i*th raw and *j*th column of the matrix *T*. The value of *r_i_* represents the total effects that *HSS_i_* has on the other *HSS*. The value of *c_j_* indicates the total effects that all the other HSS have on *HSS_j_*. For *i* = *j*, the (*r_i_* + *c_i_*) shows how important the *HSS_i_* is; it reflects the power of influences given and received by the *HSS_i_*, whereas (*ri* − *ci*) provides information about its net influence on the other *HSS*. If (*r_i_*− *c_i_*) > 0, *HSS_i_* has a net influence on the other *HSS* and is classified in the cause group; if (*r_i_*− *c_i_*) < 0, *HSS_i_* is influenced by the other HSS and is classified in the effect group. The influential relation map is produced by plotting (*r_i_* + *c_i_*) and (*r_i_*− *c_i_*) values for each *HSS_i_* on horizontal and vertical axis respectively.

## 4. Application of the Proposed Method: Case of Morocco

In this section, we present an application of the proposed mixed-method 5W2D for HSS analysis in the Moroccan context. The Moroccan health system knows the interventions of a multitude of stakeholders [48]. This makes it difficult to properly determine everyone’s roles and responsibilities in the QC.

The health system in Morocco is organized around two main sectors: a public one and a private one. The first is composed of healthcare services of the Ministry of Health, Defense department, and local governments. The second incorporates the private for-profit sub-sector, which includes hospital clinics, dental surgery, pharmacies, etc., and the private not-for-profit sub-sector made up of healthcare services of the National Fund for Social Security, the Mutuals and the National Fund of Social Welfare Bodies, the Moroccan Red Crescent, and non-governmental organizations.

Moroccan people’s expectations in terms of healthcare can be summed up in the assurance of universal access to high quality healthcare services (http://conference2013.sante.gov.ma/DocumentsConf/LivreBlanc.pdf). Thus, the Ministry of Health’s new strategy (Ministry of Health, (2018), «Plan Santé 2025», https://www.sante.gov.ma/Pages/actualites.aspx?IDActu=276) considers QC as an important challenge and puts it at the forefront of concerns. To meet this challenge, this strategy relies on the participation and commitment of HSS.

### 4.1. Preliminary List of HSS

**Figure 4:**
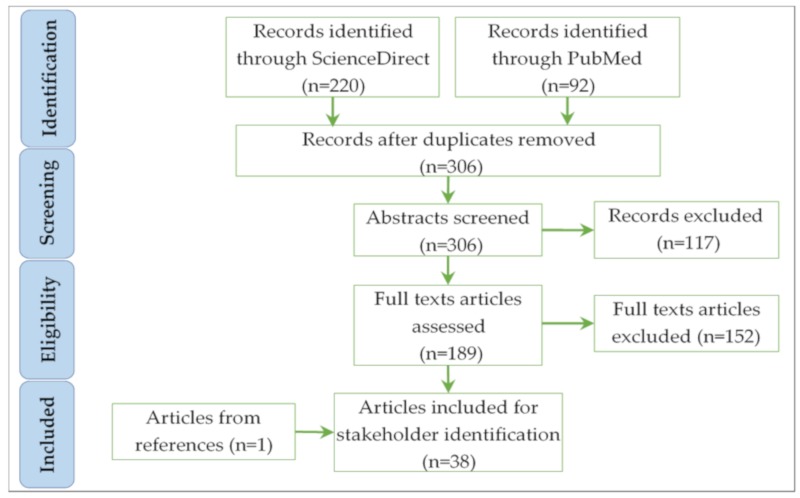
The paper selection process.

To establish the preliminary list of HSS, a systematic literature review was conducted on the Sciencedirect and PubMed databases, using *stakeholders* and *health system* as keywords, over the period from January 2015 to April 2019. A total of 306 papers were identified. After reviewing the publications’ titles and abstracts, 189 papers were selected for full reading. The selection criteria were based on papers’ objectives aiming at identifying HSS, or papers’ methods that rely on HSS opinions to address the research issue. Papers that have used the word stakeholders in an abstract way, without detailing the stakeholders’ profiles, were excluded. By doing so, 38 articles were retained (Figure 4).

The review has led to different lists of HSS. In some cases, the reasons behind the involvement of each stakeholder were not clear. The synthesis of these studies allowed for establishing a preliminary list of the main groups of HSS (Table 2). Some HSS groups are included in several papers and others are only mentioned in few papers. To avoid redundancies, we have presented the consolidated results of the main HSS groups provided by the examined papers.

### 4.2. List of HSS in Morocco

The preliminary list of HSS groups generated by the literature review was adapted to the Moroccan context using the 5W questions to investigate for correspondents in each group. What is the subject under study? Who are the involved actors? Why were they included? Where are they involved (strategic, tactical, or operational level)? The When question was considered less important for this identification phase. Answers to the retained questions were obtained through the applicable regulations, the analysis of the Ministry of Health’s reports (http://conference2013.sante.gov.ma/DocumentsConf/LivreBlanc.pdf), (http://conference2013.sante.gov.ma/DocumentsConf/intidarate%20francais.pdf), and the consultation of the “partnership” tab of its website (https://www.sante.gov.ma/Partenariat/Pages/default0.aspx) (Table 3).

### 4.3. Prioritization of HSS

Delphi method was deployed to identify further HSS and prioritize them. In this matter, five experts were contacted from the Regional Hospital Center of Fez. The latter have recommended four other experts to be included in the panel (snowball sampling). To increase the panel size, we looked for experienced profiles in the health field making use of professional and scientific social networks (i.e., linkedin, ResearchGate). Hence, 22 people were identified and approached through emails explaining the objective of the study. In feedback, seven expressed their agreement to be part of the panel (Table 4).

All participants did not add any other HSS. They judged the established list to contain all the HSS in the health system. The experts’ responses related to HSS prioritization were analyzed using IBM SPSS Statistics 23 software (New York, NY, USA). The first Delphi round indicated values of *W* less than 0.7. Accordingly, the questionnaire was sent back a second and third time to each expert for new ratings, accompanied by his or her own rating in the previous rounds and the consolidated result of the panel’s responses. The third round showed an increase of *W*, which exceeded 0.7, indicating a better degree of consensus (Table 5).

**Figure 5:**
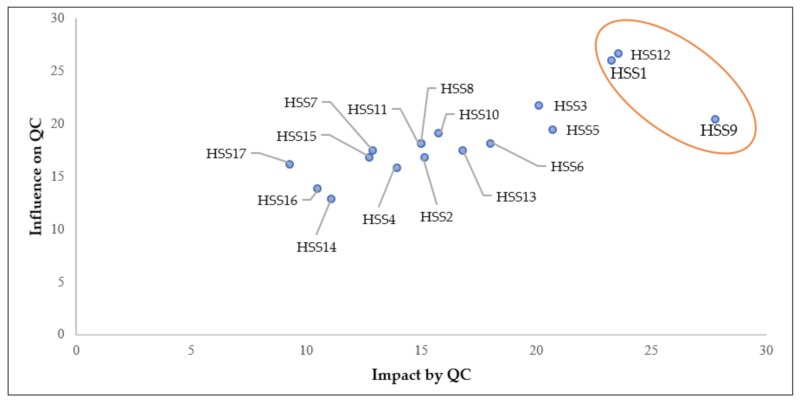
Positions of HSS according to their influence on and impact by QC.

Figure 5 shows the position of each HSS in two axes, its influence on and by impact QC. It appears that three HSS have the highest scores: HSS1, HSS12, and HSS9. Health personnel (HSS12) and government (HSS1) have the most recognized influence on QC, whereas patients and their families (HSS9) are the most affected by QC. However, it should be noted that the obtained prioritization is not the right or the correct one, but rather the one that gave an acceptable level of consensus among experts.

### 4.4. Analysis of Interrelationships between the HSS

For the purpose of determining and analyzing interrelationships between HSS, three experts were asked to complete the individual direct-relation matrix *D_e_*. The three experts belong to the Ministry of Health, Fez Regional Hospital, and the Moroccan Association for Consumer Protection and Guidance with 12, 17 and 13 years of experience respectively. The direct-relation average matrix *D* was obtained by applying Equations (3) and (4) (Table 6). It indicates the influence of HSS in the first column on those in the first row. For example, HSS1 (government) has a strong influence on HSS9 (patients and their families). The diagonal values are null, indicating that there is no influence between the same HSS.

The normalized direct-relation matrix *M* was calculated from the direct-influence matrix *D*, using Equations (5) and (6) (Table 7).

The total-relation matrix *T* was developed using Equation (7) (Table 8). In order to identify the most significant relationships, a threshold limit of influence *α* is set, which is the arithmetic mean value of the matrix *T* [41]. This calculation allows the elimination of minor effects; only values of *T* that are above the threshold *α* = 0.0272 are retained, and these correspond to the most significant influence relationships. These values are highlighted and shown in Table 8.

We calculated the sums of rows *r_i_* and columns *c_i_* of the matrix *T* for each *HSS_i_* using Equations (8) and (9) (Table 9). The (*r_i_* + *c_i_*) and (*ri* − *ci*) are used to illustrate the importance of *HSS* and to classify them into cause and effect groups. All the *HSS* with positive (*ri* − *ci*) values are in the cause group, whereas *HSS* with negative values are in the effect group. The three highest values of (*r_i_* + *c_i_*) correspond to patients and their families (HSS9), government (HSS1), and health personnel (HSS12), indicating that they are the most important in the system. The highest value of (*r_i_* − *c_i_*) matches the government, while the lowest one matches the patients and their families and health personnel. This means that the government significantly affects the other HSS, whilst the patients and their families as well as health personnel are highly influenced by all the other HSS.

**Figure 6:**
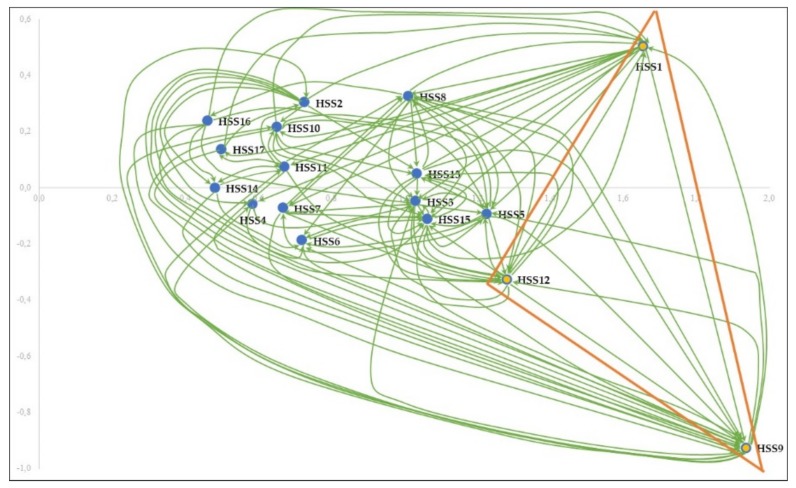
Influential relation map.

On the light of the results acquired in Table 8 and Table 9, an influential relation map is created (Figure 6). It shows significant influences given and received by each HSS. On the horizontal axis of the graph, we presented the degree of prominence (*r_i_* + *c_i_*) and on the vertical axis, the cause degree (*ri* − *ci*). The arrows indicate relationships that exceed the threshold value. It can be seen from the Figure 6 that the government (HSS1) has the highest cause effect value, while patients and their families (HSS9) and health personnel (HSS12) are the most influenced. Thereby, it can be concluded that these three HSS are the critical ones in the health system.

## 5. Discussion

Health systems are considered to be complex environments which necessitate appropriate methods to manage them [83]. This is mainly because of the several players involved in health services provision. The main objective of this paper was to identify and prioritize the HSS involved in QC. The analysis was carried out by the methodological framework 5W2D, which combines a systematic literature review, the 5W questioning technique, the Delphi method, and the DEMATEL technique. The findings confirm the existence of various HSS, who contribute to the management, production and reception of healthcare services. It should be noted that the identified HSS and their ranking are likely to vary over time. Therefore, they have to be updated at regular intervals, taking into account changes in the environment [26].

The application of the 5W2D in the Moroccan context has allowed the identification of 17 groups of HSS involved at different levels with distinct roles and interests. They are all both influencing and influenced by QC. Nevertheless, three key HSS groups are at the core of the health system:

- *Patients:* are the most important group and the reason behind the existence of health systems as the target of healthcare activities [32]. WHO emphasizes the need to place patients and populations at the center of health systems by meeting their needs and expectations [84]. The results of this study clearly showed that the action of all HSS has an impact on patients and, therefore, on the QC the way they perceive it. Similarly, patients influence QC because the care process is initiated at the patient level; patients are who decide when and where to seek care, and to continue or stop it [85]. Patients and populations also play the role of contributors to finance the health system through taxes and social security. Furthermore, the availability and access to information has radically changed the position of patients, who are no longer mere consumers of care, directed and guided by their physicians, but have become aware and able to make choices and decisions [86]. Another way that patients can influence QC is through satisfaction surveys, which are the most widely considered indicators in QC assessment. In this regard, some studies considered patients’ experience and involvement as an opportunity to improve healthcare services and bring new innovative ideas in delivering care [86].
- *Health personnel*: are the providers of healthcare services and are involved throughout the care process. This HSS group includes medical, paramedical, administrative, and other health workers, all of whom are the point of contact with patients and largely influence their perception of received care. Also, health personnel play a very potential role in QC through professional skills (effectiveness and safety of care), respect of patients’ preferences and values (patient-centeredness), optimal use of available resources (efficiency), etc. [2].
- *Government*: Through the Ministry of Health and other ministries of Finance, Education, Interior, Agriculture and Transport, the government has a significant impact on QC. The health system governance is carried out by the government, whose action is materialized by the strategies and policies it implements, as well as the funds it provides to the health system for its functioning, improvement, and innovation. The government plays a leadership role in the regulation, monitoring, and improvement of QC [87]. Generally speaking, governments play a crucial role in health development, through strengthening health systems and generating human and financial resources, to achieve objectives of improving health, efficiency and equity in health care financing [88].

The differences indicated across the key stakeholders’ roles, are noted also in their understanding and expectations associated with QC. Patients assign great importance to effective and easily accessible services, which are provided by experienced and helpful caregivers in clean and safe environments [89]. Health personnel require good working conditions and fair division of the workload. Their perception of QC involves also the availability of resources to provide care as per established practices [57]. For the government, QC implies optimal use of resources in providing essential care to a large proportion of the population. Thus, the efficiency and the accessibility of health services are placed at the central level [90]. Consequently, QC assessment and improvement need inclusive and integrated strategies, taking into consideration the perspectives of key HSS, who should be kept involved in all phases from conception to implementation.

## 6. Conclusions

Health systems are genuine crossroads where several stakeholders meet, whose roles and expectations are different. It is no longer a question of considering only the interests of patients to ensure good QC, but rather the perspectives of other HSS. However, health systems involve various groups of HSS; it is difficult to satisfy all stakeholders simultaneously since their interests are conflicting and the resources are limited. Hence, it is necessary to focus on the most critical HSS in the QC. This paper has proposed a methodological framework for identifying HSS, hierarchizing them according to their importance in QC, and analyzing their interrelationships. The proposed method is called 5W2D, which combines three basic methods: 5W, Delphi, and DEMATEL. It successfully identified HSS by applying the 5W questioning technique, hierarchized them by employing the Delphi method, and analyzed their interrelationships applying the DEMATEL technique. The results of its application in the Moroccan context reveal that three HSS are the most critical with regard to QC: patients and their families, health personnel, and the government. These key HSS have conflicting interests which are challenging and demanding in terms of finding trade-offs. For this very purpose, integrative models for understanding, assessing, and improving QC are needed, involving key HSS and taking into consideration their respective roles and expectations.

## Figures and Tables

**Table 1:** Dimensions of quality of care.

Dimension	Definition	Observable Indicators
(1) Effectiveness	Health care is based on current scientific knowledge and provided to those in need.	- Healthcare is based on current scientific knowledge (compliance with standards, compliance with recommendations, etc.). - Healthcare is provided to those who need it (targeting). - Healthcare achieves the desired outcomes.
(2) Safety	The extent to which health care processes avoid and prevent adverse events.	- Healthcare is provided in a way that reduces the occurrence of sentinel events (transfusion-related accidents, surgery on the wrong person, forgetting foreign objects in the patient’s body, etc.). - Measures are taken to minimize surgical and post-operative complications. - Healthcare is provided in a way that prevents the acquisition of nosocomial infections.
(3) Efficiency	Health care is produced with optimal use of available resources to achieve the best results.	- Resources are used in a rational way. - Activities with no added value are reduced to the minimum or even eliminated. - Waste is reduced by effective recycling.
(4) Accessibility	The availability of healthcare services and the ease with which they are reached (physical, financial, psychological accessibility).	- Medical resources are available. - Difficulties of geographical access to healthcare are reduced. - The burden to healthcare costs are reduced. - The time allocated to making appointments and waiting for care is short.
(5) Patient-leftedness	The extent to which healthcare delivery takes into account patient preferences, values and choices.	- Patients are informed about their health conditions. - Patients have the opportunity to communicate with their doctors. - Patients are involved in decision-making processes. - Healthcare is provided with respect for patients, their preferences and cultures. - Healthcare is coordinated and integrated (patient transitions between care units and between health facilities are managed effectively).
(6) Equity	Services quality does not vary because of patients’ personal characteristics such as gender, ethnicity, geographical location and socio-economic status.	- The health system guarantees services to the entire population. - Disparities in the provision of healthcare between groups in society are very small or even absent (rich/poor, rural/urban areas, etc.). - Healthcare services are provided according to patients’ needs and not to their personal characteristics.

**Table 2:** Selected publications on HSS.

**Ref**	**Objective**	**Country**	**Main HSS Groups**
[49]	Examine stakeholders’ roles to facilitate access to essential medicines.	Australia	(1) Government: ministries of health, education, transport, agriculture, industry, etc. (2) Political parties (3) Local authorities (4) Regional and provincial directorates of ministries in charge of health, education, transport, etc. (5) Health personnel: physicians, nurses, pharmacists, etc. (6) Health personnel representatives: trade unions, etc. (7) Patients and their families (8) Patient advocates (9) Health insurance bodies (10) Non-governmental organizations and the not-for-profit sector (11) United Nations agencies (12) Health facilities (13) Healthcare industry and professional associations: product suppliers (drugs, equipment, software, etc.) subcontractors (transportation, food service, etc.) (14) Health researchers: universities, laboratories, learned societies, etc. (15) Shareholders (for private healthcare facilities) (16) Employers (17) Media
[50]	Mapping challenges related to the adoption of Artificial Intelligence in the public health sector.	China	
[51]	Exploring stakeholders’ perspectives on public-private partnership in health service delivery in the Pakistani context.	Pakistan	
[52]	Studying the feasibility of integrating traditional birth attendants into maternal mental healthcare.	Kenya	
[53]	Examine the openness of health policies in terms of integrating the employees voice.	England	
[54]	Analysis of stakeholders’ views on proposed solutions to improve the performance of the Quebec health system.	Canada	
[55]	Proposition of a framework to guide the collection, planning of the implementation and use of Patient-Reported Outcome Measures.	USA	
[56]	Identification of barriers and strategies for implementing psychological interventions for older adults with cancer.	USA	
[57]	Modeling the nurse-to-patient assignment problem in home care under continuity of care while taking into consideration stakeholders’ perspectives.	Italy	
[58]	Discussing risks and legal implications of using modern telemedicine systems.	Italy	
[59]	Formulation and prioritization of strategies for the implementation of Shared Decision Making, based on the identification of obstacles to change.	Netherlands	
[60]	Establishment of guidelines for the development of radiation oncology nomenclature for clinical trials, data sharing initiatives, and population-based studies.	USA	
[61]	Description of a blueprint for the use of participatory design in the design of community pharmacy interventions.	USA	
[29]	Identification of challenges and opportunities for the implementation of regional health systems.	Singapore	
[62]	Analysis of health information technology safety assurance practices.	England	
[30]	Stakeholder analysis to identify a planning group for the development of community pharmacy service to prevent cardiovascular disease.	Australia	
[63]	Development of an innovative system of structured incentives model in an academic health sciences center, linking distribution of government payments to quality and performance outcomes.	Canada	
[64]	Identification of challenges in the implementation of non-invasive prenatal testing for fetal aneuploidy.	Netherlands	
[65]	Identification of factors promoting or hindering the sustainability of the implementation of knowledge implementation strategies in low- and middle-income contexts.	Vietnam	
[66]	Analysis of stakeholders’ opinions on the benefits and risks of data sharing in multicenter comparative efficacy research studies.	USA	
[19]	Studying stakeholders’ understanding of engagement in care improvement initiatives.	Canada	
[67]	Evaluation of the value and potential of drug repositioning.	European Union	
[68]	Analysis of stakeholders’ opinions on the Redispensing of medicines unused by patients, and their reuse by other patients.	Netherlands	
[31]	Engaging stakeholders to adapt patient-centered home care tenets for veterans to the needs of women veterans.	USA	
[69]	Identification of key determinants of practice influencing the implementation of community pharmacy services in the primary care network.	Australia	
[70]	Identification of the main problems and potential solutions to the implementation of national strategies for childhood cancer in Latin America.	Latin America	
[71]	Improving coordination between health care facilities in patient transition by identifying factors that lead to poor clinical outcomes.	USA	
[72]	Analysis of the views of different stakeholders on public engagement in the reconfiguration of emergency care systems.	Ireland	
[73]	Description of the current status of the use of electronic personal health records and identification of facilitators and barriers to their adoption.	Canada	
[74]	Analyzing stakeholder perspectives and participation in the health system change process that led to the introduction of the first Nurse Practitioner-Led clinics in Ontario.	Canada	
[75]	Analysis of stakeholders’ views on the European Union’s cross-border healthcare directive.	European Union	
[76]	Determination of the health benefits package to be included in universal health coverage that is acceptable and sustainable to stakeholders.	Lebanon	
[77]	Identification and prioritization of gaps between current scientific evidence and the actual care provided, as well as opportunities to improve the QC.	Canada	
[78]	Discussion and analysis of the reform on the organization, financing and planning of medical workforce education in England during the Conservative and Liberal Democrat coalition government’s time in office.	England	
[79]	Establishment of a comprehensive set of design requirements for digital reablement system to increase the home autonomy of older people.	England	
[80]	Proposal and development of a decentralized information system for collaboration purposes linking medical providers, paramedical providers and patients for personalized quality services.	Greece	
[81]	Understanding and analyzing facilitators and barriers to the adoption and use of clinical information systems in intensive care units.	USA	
[82]	Understanding and examining the manner in which function the networks managing health-care waste.	Palestine	

**Table 3:** Identification of HSS in Morocco.

What		
Identification of Organizations, Institutions, Individuals and Groups of Individuals Who Have an Influence or are Influenced by Health System Actions in Morocco.		
Who	Why	Where
Government: Ministries of Health, Economy and Finance, Interior, National Defense, Transport, Education, Agriculture, etc.	Responsible for the development and implementation of national health strategies. It defines the budget allocated to the implementation of health programs and ensures its monitoring and control. The government is accountable for opening up the isolated rural areas through its policies of road network development and transportation. It is also responsible for education and scientific research development strategies.	Strategic
Parliament	It is the legislative institution that passes laws, oversees government actions, and evaluates public policies, particularly those related to the health sector.	Strategic
Regional and Provincial Directorates of Ministries	Responsible for the implementation of national policies in their territories.	Tactical
Local authorities: Regions, Wilayas, prefectures, provinces and municipalities	Participate in the regional and local implementation of national policies and strategies in the health, education, and transportation sectors, etc. They ensure the diagnosis of health and hygiene needs and the maintenance of rural roads. They also manage urban transportation, patients and deceased transportation, etc.	Tactical and operational
Public and private healthcare facilities: Basic health care centers network, hospital networks, hospital clinics, hemodialysis centers, laboratories, pharmacies, etc.	Provide preventive, curative and promotional care services (consultations, nursing care, chronic disease follow-up, etc.). They also contribute to medical and pharmaceutical training activities.	Operational
Health insurance bodies: National Agency of Health Insurance (NAHI), Moroccan Health Insurance Fund (MHIF), National Fund for Social Security (NFSS), private supplementary health insurance companies.	The NAHI ensures the proper functioning of the medical coverage system by providing technical support for the obligatory health insurance. It also manages the regime of medical aid to poor populations. The MHIF manages the obligatory health insurance for civil servants and students. The NFSS manages the obligatory health insurance for employees of private enterprises and self-employed persons.	Strategic, tactical, and operational
Training and research institutions: Universities, Faculties of Medicine and Pharmacy, Health Career Training Institutes, National School of Public Health, Pasteur Institute of Morocco, Learned societies, etc.	Provide initial and in-service training in various medical, managerial and technical disciplines. They also develop research in health sciences.	Tactical and operational
International health organizations: WHO, UNICEF, UNAIDS, World Bank, etc.	Provide technical and financial assistance to the State for the development of health policies and the implementation of related programs.	Strategic Supranational
Patients and their families	Patients are the beneficiaries of healthcare services, but also any person acting on their behalf, in particular their families.	Operational
Patient advocates: consumer protection associations.	Promote consumers’ right to have access to clear, objective and fair information in particular with regard to service prices and safety.	Tactical and operational
Civil society organizations: NGOs and private not-for-profit entities working in the health field.	Help to reach disadvantaged populations and raise awareness of public health issues.	Tactical and operational
Health personnel: physicians, biologists, pharmacists, dentists, nurses, medical assistants, and administrative staff	Physicians are responsible, each within their respective areas of competence, for diagnosis and care, supervision of trainee physicians, prevention and health education. Nurses carry out medical prescriptions, provide nursing care and prepare the necessary equipment and products for medical analysis and care. The administrative staff is responsible for the management of administrative affairs.	Operational
Health personnel representatives: Order of Physicians, Order of Dentists, Order of Pharmacists, Trade unions, etc.	Professional Orders represent the medical professions vis-a-vis the government, participate in the development and implementation of health policy and defend the moral and professional interests of the medical/pharmaceutical profession. Trade unions protect the economic, industrial, commercial and professional interests of their members.	Tactical and operational
National consultation institutions: Economic, Social and Environmental Council (ESEC), National Observatory for Human Development (NOHD), National Human Rights Council (NHRC), etc.	The ESEC provides advisory services to the government on social and environmental policies. The NOHD analyses and evaluates the impact of human development programs. The NHRC sees to the observation, surveillance and monitoring of human rights.	Strategic
Healthcare industry: manufacturers, wholesalers, distributors and subcontractors	Responsible for product development, production, and distribution. They provide healthcare facilities with goods (medicines, medical supplies, equipment, etc.) and services (IT, maintenance, food services, cleaning, etc.), that are necessary for their operations and the production of care.	Tactical and operational
Employers	Provide workers with a healthy and safe working environment. They are responsible for implementing appropriate environmental and health protection measures against pollution generated by industrial activities.	Operational
Media	Contribute to the promotion of health information and public awareness.	Tactical and operational

**Table 4:** Experts included in the Delphi survey.

N°	Affiliation	Position	Years of Experience	Degree	City
1	Regional Hospital	Quality manager	13	Doctor	Fez
2		Head of Medical Affairs Department	17	Doctor	Fez
3		Administrator	10	Master	Fez
4		Quality manager	10	Maser	Tangier
5		Purchasing manager	16	Master	Fez
6		Head of the Nursing Care Unit	10	Technician	Fez
7	Ministry of Health	Administrator	12	Master	Rabat
8	University	Health researcher	10	Doctor	Casablanca
9		Health researcher	11	Doctor	Tangier
10	Moroccan Association for Consumer Protection and Guidance	Member of the Association	13	Master	Kenitra
11	University Hospital	Head of Performance Evaluation Department	24	Master	Rabat
12	Prefectural Hospital	Quality manager	15	Doctor	Casablanca
13	Pasteur Institute	Health researcher	11	Doctor	Casablanca
14	Regional Oncology Centre	Administrator	12	Master	Meknes
15		Head of Medical Affairs Department	23	Doctor	Meknes
16		Administrator	12	Master	Meknes

**Table 5:** Results of the three Delphi rounds of HSS prioritization.

HSS	Influence on QC						Impact by QC					
	First Round		Second Round		Third Round		First Round		Second Round		Third Round	
	Mean(*M_k_*)	Standard Deviation	Mean(*M_k_*)	Standard Deviation	Mean(*M_k_*)	Standard Deviation	Mean(*N_k_*)	Standard Deviation	Mean(*N_k_*)	Standard Deviation	Mean(*N_k_*)	Standard Deviation
HSS1: Government	25.25	4.18	25.33	3.27	26.13	2.80	22.83	3.46	23.83	3.04	23.20	2.73
HSS2: Parliament	18.58	3.40	17.13	3.31	16.80	1.90	15.25	6.22	16.67	4.42	15.07	4.82
HSS3: Regional and Provincial Directorates of Ministries of Health, Transport, Interior, etc.	22.33	3.20	22.53	2.56	21.80	2.18	19.67	4.19	20.33	4.01	20.13	3.60
HSS4: Local authorities	16.17	2.92	16.00	1.51	16.00	1.51	14.17	5.25	15.17	3.30	13.87	4.60
HSS5: Public and private healthcare facilities	19.00	2.95	19.53	2.59	19.47	2.00	20.75	4.45	20.08	3.50	20.67	3.44
HSS6: Health insurance bodies	17.50	3.18	17.93	1.94	18.27	1.75	19.00	4.16	18.42	3.65	18.00	3.27
HSS7: Training and research institutions	17.33	3.17	17.80	1.70	17.47	1.81	13.42	4.34	13.33	4.12	12.87	3.83
HSS8: International health organizations	20.00	3.64	18.67	2.35	18.27	2.09	16.25	4.94	16.00	4.71	15.00	3.72
HSS9: Patients and their families	21.92	3.94	19.87	2.26	20.60	2.23	26.83	2.92	27.92	2.43	27.80	2.31
HSS10: Patient advocates	20.00	2.95	17.33	2.87	19.07	1.71	15.50	2.94	14.75	2.18	15.80	2.04
HSS11: Civil society organizations	18.33	2.10	18.00	1.46	18.00	1.46	14.17	4.45	14.92	4.25	14.93	3.47
HSS12: Health personnel	26.42	3.82	25.73	2.94	26.53	2.80	21.50	4.42	23.92	4.52	23.60	3.87
HSS13: Health personnel representatives	18.75	3.02	17.53	2.20	17.53	2.20	17.00	3.79	17.67	3.20	16.80	2.86
HSS14: Employers	14.00	3.16	13.40	1.96	12.87	1.92	12.00	4.00	11.50	3.21	11.07	2.81
HSS15: Healthcare industry	17.08	2.75	16.80	2.01	16.93	2.02	13.00	2.63	13.25	2.30	12.73	2.22
HSS16: National consultation institutions	14.83	3.97	13.80	2.54	13.80	2.54	11.17	4.34	11.00	4.11	10.53	3.66
HSS17: Media	16.50	3.61	16.27	2.22	16.27	2.12	9.58	4.25	9.42	3.82	9.33	3.22
Kendall’s *W* coefficient of concordance	0.468		0.626		0.705		0.560		0.665		0.708	

**Table 6:** Direct-relation average matrix *D.*

	HSS1	HSS2	HSS3	HSS4	HSS5	HSS6	HSS7	HSS8	HSS9	HSS10	HSS11	HSS12	HSS13,	HSS14	HSS15	HSS16,	HSS17
HSS1	0.0000	0.6667	4.0000	2.0000	3.0000	3.0000	3.6667	1.3333	4.0000	1.3333	2.3333	4.0000	2.6667	3.3333	3.0000	0.6667	2.0000
HSS2	2.3333	0.0000	0.6667	1.3333	1.0000	0.6667	0.6667	0.3333	1.6667	1.6667	1.3333	1.6667	1.0000	2.0000	0.6667	1.3333	0.3333
HSS3	0.6667	0.0000	0.0000	0.6667	4.0000	0.0000	1.3333	0.3333	4.0000	0.3333	1.0000	2.6667	0.6667	0.0000	1.6667	0.0000	0.3333
HSS4	0.3333	0.0000	1.3333	0.0000	0.6667	0.0000	0.3333	0.0000	3.6667	0.0000	1.3333	0.6667	0.0000	1.0000	0.0000	0.0000	0.3333
HSS5	0.6667	0.0000	1.3333	0.3333	0.0000	1.3333	2.0000	1.6667	4.0000	0.3333	0.6667	4.0000	1.6667	0.0000	2.0000	0.0000	0.6667
HSS6	0.3333	0.0000	0.0000	0.0000	1.0000	0.0000	0.0000	0.6667	4.0000	0.0000	0.0000	0.3333	0.3333	0.0000	2.6667	0.0000	0.0000
HSS7	0.6667	0.0000	0.0000	0.0000	0.0000	0.3333	0.0000	1.6667	3.6667	0.0000	0.0000	1.3333	0.3333	0.0000	2.3333	0.0000	0.0000
HSS8	2.6667	0.3333	1.3333	0.3333	2.0000	1.3333	1.3333	0.0000	3.0000	0.0000	1.0000	3.3333	2.3333	0.0000	3.0000	1.3333	0.3333
HSS9	2.3333	2.0000	1.6667	2.3333	1.0000	0.3333	0.6667	0.3333	0.0000	1.3333	1.3333	2.3333	0.6667	0.0000	0.6667	0.6667	0.3333
HSS10	1.6667	0.6667	2.3333	0.3333	2.3333	0.0000	0.0000	0.3333	2.0000	0.0000	0.3333	1.0000	1.6667	0.3333	1.3333	0.0000	0.6667
HSS11	0.6667	0.6667	0.0000	0.6667	0.0000	0.0000	0.0000	1.3333	3.6667	1.3333	0.0000	1.3333	0.6667	1.6667	0.0000	0.0000	1.6667
HSS12	0.6667	0.0000	1.3333	0.0000	1.6667	1.6667	1.0000	1.6667	4.0000	0.0000	0.3333	0.0000	3.3333	0.0000	1.3333	0.0000	0.0000
HSS13	2.3333	0.3333	1.6667	0.3333	2.0000	0.3333	0.6667	1.6667	3.3333	0.3333	0.3333	3.6667	0.0000	0.0000	1.6667	0.0000	0.0000
HSS14	0.6667	0.3333	0.0000	1.0000	0.0000	3.0000	0.0000	0.0000	4.0000	0.0000	0.0000	0.0000	0.0000	0.0000	0.0000	0.0000	0.0000
HSS15	2.0000	0.3333	1.6667	0.0000	2.6667	2.6667	0.6667	0.6667	4.0000	0.0000	0.0000	0.6667	1.3000	0.0000	0.0000	0.0000	0.0000
HSS16	1.3333	0.0000	1.3333	1.0000	1.6667	1.3333	0.3333	0.0000	2.3333	0.3333	0.0000	0.6667	0.3333	1.3333	0.6667	0.0000	0.0000
HSS17	1.6667	1.6667	0.3333	0.6667	1.0000	0.6667	0.0000	0.0000	2.6667	0.6667	0.3333	0.6667	0.3333	0.0000	0.3333	0.0000	0.0000

**Table 7:** Normalized direct-relation matrix *M.*

	HSS1	HSS2	HSS3	HSS4	HSS5	HSS6	HSS7	HSS8	HSS9	HSS10	HSS11	HSS12	HSS13	HSS14	HSS15	HSS16	HSS17
HSS1	0.0000	0.0123	0.0741	0.0370	0.0556	0.0556	0.0679	0.0247	0.0741	0.0247	0.0432	0.0741	0.0494	0.0617	0.0556	0.0123	0.0370
HSS2	0.0432	0.0000	0.0123	0.0247	0.0185	0.0123	0.0123	0.0062	0.0309	0.0309	0.0247	0.0309	0.0185	0.0370	0.0123	0.0247	0.0062
HSS3	0.0123	0.0000	0.0000	0.0123	0.0741	0.0000	0.0247	0.0062	0.0741	0.0062	0.0185	0.0494	0.0123	0.0000	0.0309	0.0000	0.0062
HSS4	0.0062	0.0000	0.0247	0.0000	0.0123	0.0000	0.0062	0.0000	0.0679	0.0000	0.0247	0.0123	0.0000	0.0185	0.0000	0.0000	0.0062
HSS5	0.0123	0.0000	0.0247	0.0062	0.0000	0.0247	0.0370	0.0309	0.0741	0.0062	0.0123	0.0741	0.0309	0.0000	0.0370	0.0000	0.0123
HSS6	0.0062	0.0000	0.0000	0.0000	0.0185	0.0000	0.0000	0.0123	0.0741	0.0000	0.0000	0.0062	0.0062	0.0000	0.0494	0.0000	0.0000
HSS7	0.0123	0.0000	0.0000	0.0000	0.0000	0.0062	0.0000	0.0309	0.0679	0.0000	0.0000	0.0247	0.0062	0.0000	0.0432	0.0000	0.0000
HSS8	0.0494	0.0062	0.0247	0.0062	0.0370	0.0247	0.0247	0.0000	0.0556	0.0000	0.0185	0.0617	0.0432	0.0000	0.0556	0.0247	0.0062
HSS9	0.0432	0.0370	0.0309	0.0432	0.0185	0.0062	0.0123	0.0062	0.0000	0.0247	0.0247	0.0432	0.0123	0.0000	0.0123	0.0123	0.0062
HSS10	0.0309	0.0123	0.0432	0.0062	0.0432	0.0000	0.0000	0.0062	0.0370	0.0000	0.0062	0.0185	0.0309	0.0062	0.0247	0.0000	0.0123
HSS11	0.0123	0.0123	0.0000	0.0123	0.0000	0.0000	0.0000	0.0247	0.0679	0.0247	0.0000	0.0247	0.0123	0.0309	0.0000	0.0000	0.0309
HSS12	0.0123	0.0000	0.0247	0.0000	0.0309	0.0309	0.0185	0.0309	0.0741	0.0000	0.0062	0.0000	0.0617	0.0000	0.0247	0.0000	0.0000
HSS13	0.0432	0.0062	0.0309	0.0062	0.0370	0.0062	0.0123	0.0309	0.0617	0.0062	0.0062	0.0679	0.0000	0.0000	0.0309	0.0000	0.0000
HSS14	0.0123	0.0062	0.0000	0.0185	0.0000	0.0556	0.0000	0.0000	0.0741	0.0000	0.0000	0.0000	0.0000	0.0000	0.0000	0.0000	0.0000
HSS15	0.0370	0.0062	0.0309	0.0000	0.0494	0.0494	0.0123	0.0123	0.0741	0.0000	0.0000	0.0123	0.0241	0.0000	0.0000	0.0000	0.0000
HSS16	0.0247	0.0000	0.0247	0.0185	0.0309	0.0247	0.0062	0.0000	0.0432	0.0062	0.0000	0.0123	0.0062	0.0247	0.0123	0.0000	0.0000
HSS17	0.0309	0.0309	0.0062	0.0123	0.0185	0.0123	0.0000	0.0000	0.0494	0.0123	0.0062	0.0123	0.0062	0.0000	0.0062	0.0000	0.0000

**Table 8:** Total-relation matrix T.

	HSS1	HSS2	HSS3	HSS4	HSS5	HSS6	HSS7	HSS8	HSS9	HSS10	HSS11	HSS12	HSS13	HSS14	HSS15	HSS16	HSS17
HSS1	0.0244	0.0217	0.0940	0.0497	0.0829	0.0740	0.0822	0.0409	0.1373	0.0329	0.0545	0.1073	0.0697	0.0672	0.0804	0.0159	0.0432
HSS2	0.0538	0.0048	0.0253	0.0322	0.0322	0.0230	0.0209	0.0136	0.0607	0.0354	0.0313	0.0473	0.0294	0.0430	0.0243	0.0266	0.0108
HSS3	0.0240	0.0053	0.0122	0.0192	0.0851	0.0094	0.0337	0.0153	0.1010	0.0109	0.0249	0.0678	0.0245	0.0029	0.0426	0.0021	0.0100
HSS4	0.0125	0.0040	0.0299	0.0055	0.0185	0.0041	0.0101	0.0036	0.0804	0.0036	0.0285	0.0216	0.0048	0.0205	0.0050	0.0013	0.0085
HSS5	0.0268	0.0059	0.0376	0.0136	0.0165	0.0347	0.0458	0.0402	0.1053	0.0109	0.0192	0.0924	0.0444	0.0029	0.0516	0.0028	0.0155
HSS6	0.0144	0.0040	0.0076	0.0049	0.0262	0.0059	0.0048	0.0160	0.0864	0.0030	0.0039	0.0163	0.0122	0.0013	0.0548	0.0017	0.0018
HSS7	0.0214	0.0041	0.0084	0.0051	0.0093	0.0129	0.0054	0.0349	0.0825	0.0030	0.0044	0.0353	0.0139	0.0018	0.0503	0.0022	0.0019
HSS8	0.0645	0.0122	0.0418	0.0154	0.0568	0.0387	0.0373	0.0119	0.0947	0.0063	0.0270	0.0855	0.0588	0.0063	0.0725	0.0273	0.0113
HSS9	0.0534	0.0404	0.0433	0.0496	0.0333	0.0152	0.0216	0.0140	0.0304	0.0296	0.0322	0.0600	0.0243	0.0072	0.0244	0.0147	0.0110
HSS10	0.0410	0.0165	0.0540	0.0127	0.0567	0.0086	0.0092	0.0133	0.0622	0.0045	0.0128	0.0367	0.0406	0.0100	0.0357	0.0020	0.0160
HSS11	0.0228	0.0178	0.0092	0.0191	0.0095	0.0070	0.0053	0.0287	0.0855	0.0287	0.0056	0.0365	0.0202	0.0337	0.0078	0.0025	0.0333
HSS12	0.0256	0.0052	0.0361	0.0071	0.0446	0.0384	0.0268	0.0387	0.0996	0.0046	0.0126	0.0206	0.0707	0.0024	0.0382	0.0026	0.0031
HSS13	0.0557	0.0114	0.0453	0.0142	0.0536	0.0179	0.0240	0.0400	0.0927	0.0113	0.0145	0.0882	0.0154	0.0047	0.0458	0.0031	0.0046
HSS14	0.0180	0.0098	0.0055	0.0234	0.0055	0.0581	0.0032	0.0026	0.0847	0.0031	0.0040	0.0074	0.0036	0.0021	0.0061	0.0016	0.0017
HSS15	0.0477	0.0112	0.0425	0.0079	0.0625	0.0572	0.0221	0.0202	0.1000	0.0051	0.0074	0.0324	0.0343	0.0038	0.0146	0.0026	0.0040
HSS16	0.0317	0.0034	0.0329	0.0240	0.0401	0.0316	0.0126	0.0050	0.0633	0.0093	0.0051	0.0247	0.0131	0.0275	0.0211	0.0014	0.0027
HSS17	0.0385	0.0344	0.0149	0.0183	0.0271	0.0180	0.0062	0.0046	0.0653	0.0167	0.0118	0.0244	0.0134	0.0045	0.0139	0.0022	0.0032

Color: values are above the threshold 0.0272.

**Table 9:** Influences received and given by each *HSS.*

	r_i_	c_i_	r_i_ + c_i_ (Prominence Degree)	ri − ci (Cause Degree)
HSS1	1.0782	0.5760	1.6542	0.5021
HSS2	0.5146	0.2121	0.7267	0.3025
HSS3	0.4910	0.5407	1.0317	−0.0498
HSS4	0.2624	0.3219	0.5843	−0.0595
HSS5	0.5662	0.6606	1.2268	−0.0944
HSS6	0.2654	0.4546	0.7200	−0.1892
HSS7	0.2969	0.3712	0.6681	−0.0743
HSS8	0.6681	0.3435	1.0116	0.3246
HSS9	0.5047	1.4320	1.9367	−0.9272
HSS10	0.4325	0.2189	0.6515	0.2136
HSS11	0.3731	0.2998	0.6729	0.0733
HSS12	0.4769	0.8044	1.2814	−0.3275
HSS13	0.5423	0.4935	1.0358	0.0488
HSS14	0.2401	0.2418	0.4819	−0.0016
HSS15	0.4756	0.5891	1.0647	−0.1135
HSS16	0.3495	0.1126	0.4621	0.2370
HSS17	0.3176	0.1825	0.5001	0.1351
